# A Novel Approach on Deep Learning—Based Decision Support System Applying Multiple Output LSTM-Autoencoder: Focusing on Identifying Variations by PHSMs’ Effect over COVID-19 Pandemic

**DOI:** 10.3390/ijerph19116763

**Published:** 2022-06-01

**Authors:** Yong-Ju Jang, Min-Seung Kim, Chan-Ho Lee, Ji-Hye Choi, Jeong-Hee Lee, Sun-Hong Lee, Tae-Eung Sung

**Affiliations:** 1 Department of Computer Science, Graduate School, Yonsei University, Wonju 26493, Korea; lolhi@yonsei.ac.kr (Y.-J.J.); kms903@yonsei.ac.kr (M.-S.K.); chpat2785@yonsei.ac.kr (C.-H.L.); toddlf58@yonsei.ac.kr (J.-H.C.); smartbio@naver.com (J.-H.L.); bugeelee@naver.com (S.-H.L.); 2 Division of Software, Yonsei University, Wonju 26493, Korea

**Keywords:** decision support system, public health and social measures (PHSMs), deep learning, LSTM-Autoencoder, COVID-19

## Abstract

Following the outbreak of the COVID-19 pandemic, the continued emergence of major variant viruses has caused enormous damage worldwide by generating social and economic ripple effects, and the importance of PHSMs (Public Health and Social Measures) is being highlighted to cope with this severe situation. Accordingly, there has also been an increase in research related to a decision support system based on simulation approaches used as a basis for PHSMs. However, previous studies showed limitations impeding utilization as a decision support system for policy establishment and implementation, such as the failure to reflect changes in the effectiveness of PHSMs and the restriction to short-term forecasts. Therefore, this study proposes an LSTM-Autoencoder-based decision support system for establishing and implementing PHSMs. To overcome the limitations of existing studies, the proposed decision support system used a methodology for predicting the number of daily confirmed cases over multiple periods based on multiple output strategies and a methodology for rapidly identifying varies in policy effects based on anomaly detection. It was confirmed that the proposed decision support system demonstrated excellent performance compared to models used for time series analysis such as statistical models and deep learning models. In addition, we endeavored to increase the usability of the proposed decision support system by suggesting a transfer learning-based methodology that can efficiently reflect variations in policy effects. Finally, the decision support system proposed in this study provides a methodology that provides multi-period forecasts, identifying variations in policy effects, and efficiently reflects the effects of variation policies. It was intended to provide reasonable and realistic information for the establishment and implementation of PHSMs and, through this, to yield information expected to be highly useful, which had not been provided in the decision support systems presented in previous studies.

## 1. Introduction

The COVID-19 pandemic poses a critical threat to the world. COVID-19, which first appeared in Wuhan, Hubei Province, China, in December 2019, has rapidly spread worldwide [1], and the World Health Organization (WHO) declared a pandemic in March 2020. As of 31 December 2021, the cumulative number of confirmed COVID-19 cases worldwide was 288,707,020, and the cumulative number of deaths was 5,440,149, and in the Republic of Korea (hereinafter South Korea), the totals were 635,353 and 5625 during the same period [2]. Due to the COVID-19 epidemic, the global economy has contracted significantly. In the case of South Korea, the annual GDP growth rate decreased by 3% and the private consumption growth rate decreased by more than 7% in 2020 compared to 2019. This was analyzed to be the second largest economic recession since the economic crisis in 1998 [3]. In addition, the number of critically ill patients worldwide increased significantly, posing a major threat to public health systems around the world. In addition, there is a significant increase in the number of cases of critical illness worldwide, posing a great threat to the global public health system. In the case of South Korea, due to the rapid spread of the COVID-19 epidemic in the metropolitan area, as of 21 December 2021, the utilization rate of intensive care beds increased to 85.7%, and the number of people waiting to be assigned to intensive care beds increased significantly to 420 [4].

To overcome this threat, each country responded to the spread of COVID-19 by implementing various Public Health and Social Measures (PHSMs), such as Non-Pharmaceutical Interventions (NPIs) in the early stage of COVID-19 epidemic and pharmacological methods such as COVID-19 vaccines from 2021. To maximize the effectiveness of these PHSMs, it is necessary to quickly review the suitability of policies and plan the establishment of additional policies, considering various factors such as social and economic ripple effects from the implementation of PHSMs. However, it is not easy to review the suitability of currently implemented policies when factors that can change the effectiveness of a policy suddenly emerge or when several factors interact in a complex way. Therefore, this study proposes a decision support system for establishing and implementing PHSMs based on deep learning. The proposed decision support system performed deep learning-based anomaly detection and multi-step prediction on confirmed COVID-19 cases to overcome the limitations of the existing methods of policy suitability review mentioned above. This proposed decision support system is intended to provide various types of information. The main contributions of this study can be summarized as follows:

- The proposed decision support system has superior training capacity in multi-period time series prediction.
- The proposed decision support system can quickly identify variability in the effectiveness of PHSMs.
- The proposed decision support system can efficiently train the change effect when the effect of PHSMs is changed.
- The proposed decision support system provides various information as described above, helping establish and implement PHSMs.

The composition of this study is as follows. Section 2 introduces previous studies related to the current status of PHSMs and the decision support system for policy establishment. Section 3 introduces the background of the methodologies applied in this study. Section 4 introduces the dataset and algorithm structure used in this study. Section 5 introduces the experimental and verification results of the proposed model. Section 6 derives the implications of the decision support system proposed in this study. Finally, Section 7 presents a summary of this study and discusses suggestions for further studies.

## 2. Related Research

### 2.1. Public Health and Social Measures (PHSMs)

Since the global spread of COVID-19, many efforts have been made internationally to contain COVID-19. Specifically, PHSMs such as NPIs and pharmacological methods effectively controlled COVID-19 infections. Among them, NPIs, implemented by many countries from the beginning of the COVID-19 pandemic to the present, are policies to stem the epidemic by minimizing personal contact, and many countries are implementing social distancing measures as NPI.

NPIs can be classified into two strategies: suppression and mitigation. Suppression refers to a strategy to prevent interpersonal transmission by reducing the reproductive number of infectious diseases to one or less by minimizing contact between individuals by using policies such as city lockdowns [5]. Mitigation, on the other hand, is a strategy that does not completely prevent the epidemic but protects specific individuals who may develop a serious disease due to an epidemic. This aims to minimize the health threat caused by infectious diseases, reduce the peak demand for medical services, and ultimately reduce the number of confirmed cases and reproductive numbers of an epidemic through herd immunity [5]. Although there are some differences across countries, these NPIs are generally implemented according to national policy, consisting of school and workplace closures, cancellation of public events, restrictions on private gatherings, closure of public transportation, staying at home, and restrictions on movement inside and outside the country [6]. It is the first time since the H1N1 influenza epidemic in 1918 that the world has used NPIs to respond to a new epidemic on the scale of the current COVID-19 pandemic. Cities that performed NPIs in the early stages during the H1N1 influenza pandemic showed a great effect in controlling the spread of infectious diseases, such as reducing the number of confirmed cases and maintaining low mortality [5]. Based on these cases, many countries are implementing NPIs, and these have showed a significant effect in controlling the spread of COVID-19 [7,8,9,10,11]. It has been confirmed that social distancing implemented in South Korea also had a significant effect, such as reducing the reproductive number (R) to 1 or less [12].

As such, PHSMs are known to have a significant benefit in controlling the spread of COVID-19, but in practice, it is necessary to consider that the effectiveness of these policies may change continuously. This can be confirmed in the case of the South Korea, where as time passed, the time until the detection of an effect and the time for the maximum effect increased gradually, but the duration of the maximum effect appeared to decrease [13]. This means that the effect of the policy varied due to various causes, and these can be classified into two types of causes. First, the effect of the policy itself changes. Social distancing, a type of NPI, is being implemented in several countries to control the spread of COVID-19, but it is known that the effectiveness of NPIs decreases if the public’s compliance with the policy is low [14]. Second, there are cases where the effect of a policy changes due to external factors. The spread of highly contagious variants of the COVID-19 virus is known to significantly lower the infection prevention rate and the severe disease prevention rate of existing vaccines [15], and the effectiveness of NPIs can also be relatively reduced.

### 2.2. Decision Support System for PHSMs

A representative simulation model for predicting the spread of an infectious disease is the mathematical modelling of infectious disease. The mathematical modeling of infectious disease is a model that subdivides the entire population into mutually exclusive categories using ordinary differential equations [16]. The mathematical modeling of infectious disease generally uses the Susceptible-Infectious-Recovered (SIR) [17] model, and modeling is performed by adding assumptions to the SIR model according to the characteristics of the infectious disease. Cooper et al. [18] performed an analysis of the COVID-19 epidemic in various countries using the SIR model. As a result of the study, the proposed SIR model showed excellent performance and provided information related to the spread of the virus over time that could not be obtained with data alone. Basnarkov [19] proposed a Susceptible-Exposed-Asymptomatic-Infectious-Recovered (SEAIR) model. The proposed SEAIR is a model that adds a symptom compartment to the SEIR model, and the infection characteristics of COVID-19 were analyzed. Zhan et al. [20] proposed a Susceptible-Exposed-Infectious-Confirmed-Recovered (SEICR) model. The proposed model is a model that adds a Confirm compartment to the Susceptible-Exposed-Infectious-Recovered (SEIR) model, and as a result of the experiment, it successfully predicted the spread of COVID-19 in various countries and emphasized the risk of unconfirmed COVID-19 patients. Zou et al. [21] proposed the SEIR model and Susceptible-Exposed-Infectious-Quarantine-Recovered-Vaccination (SEIQRV) model that considers population movement between cities. As a result of analyzing the CFS rush period, the reproductive number decreased in the province with large-scale emigration, and the reproductive number increased in the province with large-scale immigration.

In addition, a study combining a mathematical modelling of infectious disease and a machine learning model has been proposed. Shweta et al. [22] proposed an ensemble model that combines a mathematical modelling of infectious disease and a machine learning model. The infection rate was calculated using the Susceptible-Exposed-Recovered-Fatalities (SIRF) model, and the end of the pandemic was predicted using the Naïve Bayes Classifier. Amaral et al. [23] proposed a Susceptible-Infectious-Recovered-Deceased (SIRD) model and used ANN as a methodology for rationally estimating β, which means effective transmission rate. Vega et al. [24] proposed an SIMLR model that combines the SIR model and the machine learning model. The proposed SIMLR model uses NN-CPD to estimate the change in policy and reflects this in the SIR model to predict the number of COVID-19 confirmed cases. Furthermore, it can be confirmed that many studies have combined machine learning with infectious disease mathematical models [25,26,27].

As a result of a review of previous studies on the mathematical modelling of infectious disease, it was confirmed that various models, including the SIR model, are widely used. However, it was confirmed that the more complex the characteristics of the infectious disease, the more complex the mathematical modeling of infectious disease. A complex infectious disease mathematical models is more realistic than a simple model by integrating the biological and epidemiologic information of an infectious disease, but there is a limitation in that the number of parameters to be estimated increases accordingly, increasing the uncertainty of the model [28]. To this end, research related to simulation models using machine learning and deep learning with high predictive performance instead of mathematical models with high uncertainty are increasing.

Alabdulrazzaq et al. [29] reviewed whether the Autoregressive Integrated Moving Average (ARIMA) model is suitable in a complex and dynamic situation such as COVID-19. As an experimental result, they confirmed its excellent performance. Ballı [30] predicted the number of short-term cumulative confirmed cases of COVID-19 in the world, Germany, and the United States by using various machine learning techniques, and Support Vector Machine (SVM) showed the best performance. Lounis et al. [31] predicted the number of confirmed, deaths, and recovery COVID-19 cases over 6 months using SVM and Decision Tree. Masum et al. [32] compared the prediction performance of the mathematical modeling of infectious disease, ARIMA, Long-Short Term Memory (LSTM), Bidirectional Recurrent Neural Network (Bidirectional RNN), and Gate Recurrent Unit (GRU) model to predict the cumulative confirmed cases of COVID-19. As a result of the experiment, the Bidirectional RNN showed the best performance but pointed out that there is a limit to the interpretation. Arora et al. [33] compared and analyzed the performance of Deep LSTM, Convolution LSTM, and Bidirectional LSTM models using data on the number of confirmed COVID-19 cases by state in India in a short period of time. Each model was constructed in the form of predicting the number of confirmed cases the next day, and the performance of the Bidirectional LSTM was shown to be the best among the proposed models. Dairi et al. [34] compared the prediction performance of a traditional machine learning model, a deep learning model, and a hybrid deep learning model using short-term COVID-19 statistics data from seven countries. Among the models for predicting the number of confirmed cases the next day, the hybrid model LSTM-CNN exhibited the best performance. Maaliw et al. [35] predicted the number of COVID-19 cases and deaths in the Philippines, the United States, India, and Brazil by ensembles in the ARIMA model and the Stacked LSTM model. Furthermore, it can be confirmed that many studies have utilized statistical models, machine learning, and deep learning [36,37,38,39,40].

Recently, research to predict COVID-19 using complex deep learning models has been proposed. Kim et al. [41] proposed a Hi-COVIDNet that predicts the risk of COVID-19 transmission in a target country by reflecting the complex relationship between countries. The proposed Hi-COVIDNet predicts the number of multi-period COVID-19 confirmed cases in the target country by hierarchically constructing Country-Level encoder and Continent-Level encoder using the collected Intra-Country dataset and Inter-Country dataset. Gao et al. [42] proposed Spatio-Temporal Attention Network (STAN) for predicting the COVID-19 pandemic. The proposed STAN model combines Graph Attention Network (GAT) and GRU and performs multi-period prediction using dynamic and static data as input. Zhou et al. [43] proposed an Interpretable Temporal Attention Network (ITANet) to overcome the limitations of the existing deep learning-based COVID-19 prediction. The proposed ITANet consists of an encoder–decoder structure model and a Covariate Forecasting Network (CFN) model to reflect covariates. In particular, it was shown that the importance of covariates such as government intervention can be revealed through the temporal covariate interpreter.

In reviewing these previous studies, we are able to confirm the appropriateness of a decision support system based on COVID-19 simulation using a mathematical model, machine learning, and deep learning. However, most previous studies have limitations in that they constructed a simulation-based decision support system without considering the variations in the effects of PHSMs. Most studies fail to account for the fact that the effects of PHSMs can continuously change and that the effects of policies cannot be sufficiently assumed or trained with short-term data and applied a simulation model that only assumes and performs training based on the effects of past policies. In addition, in a simulation model that assumes only the effects of past policies and trains on that basis, if the effects of policies change due to external factors, these changes cannot be reflected in the results. The information generated from such a decision support system is inappropriate to use as a basis for policy establishment and enforcement, and there is a risk of making an incorrect decision by referring to it. In addition, previous studies using machine learning and deep learning are decision support systems that predict the number of COVID-19 cases in a short period of time. This decision support system has limitations in usability from the point of view of a country that needs to establish and implement PHSMs by examining relatively long-term epidemic trends. Therefore, this study proposes a decision support system that supplements the limitations of previous studies by performing deep learning-based anomaly detection and multi-step prediction.

## 3. Background

### 3.1. Anomaly Detection in Time Series Data

Anomaly detection refers to the task of finding a pattern in data that is statistically different from data with normal characteristics [44]. The importance of abnormal data detected using anomaly detection is that it can consist of information that had not been previously discovered and can often be used as actionable information [45]. Anomaly detection is being used in various fields, such as the detection of aircraft engine rotation coupling, the detection of defects in manufacturing facilities, and the fraudulent use of credit cards [46].

Anomaly detection is mainly performed using time series data. Abnormal data in time series data can be defined by a difference from past trends or patterns over time. Previous studies related to anomaly detection in time series data were conducted based on traditional time series analysis models such as ARIMA. Recently, research is being actively conducted to perform anomaly detection using machine learning. Machine learning-based anomaly detection research can be broadly classified into three categories: supervised anomaly detection, semi-supervised anomaly detection, and unsupervised anomaly detection.

Supervised anomaly detection performs anomaly detection using a supervised learning-based machine learning algorithm when normal and abnormal samples are labeled in given time series data [45]. However, supervised anomaly detection can require a lot of time to collect abnormal samples. In addition, since the number of abnormal samples is generally smaller than that of normal samples, a class imbalance problem occurs, which renders training difficult.

Semi-supervised anomaly detection is a method designed to solve the problem of the high levels of time and expense required due to abnormal sample collection and the problem of the class imbalance that may occur in supervised anomaly detection. Semi-supervised anomaly detection generates a discriminative boundary by training with only normal samples and discriminates all samples outside the boundary as abnormal [45]. Representative algorithms that implement semi-supervised anomaly detection include One-Class SVM proposed by Manevitz et al. [47] and Deep SVDD proposed by Ruff et al. [48].

Unlike the above two methodologies, unsupervised anomaly detection performs anomaly detection on unlabeled data [45]. Supervised and semi-supervised anomaly detection methodologies require labeled data. However, data that are not actually labeled or cannot be labeled comprise the majority, and even if labeling is performed on data, this necessitates the expenditure of a lot of time and money. To this end, unsupervised anomaly detection, which performs anomaly detection on unlabeled data, has been proposed. In general, autoencoder models such as Autoencoder, Variational Autoencoder [49], Adversarial Autoencoder [50], and LSTM-Autoencoder [51] are used to perform unsupervised anomaly detection. Autoencoder is a special model that reconstructs input data. Through this process, the important characteristics of the input data are learned. Anomaly detection using an autoencoder learns important characteristics of normal samples by learning under the assumption that most of the data used for training are normal samples. In addition, the loss function-based threshold is calculated [52,53,54,55], and if there is a loss function value that exceeds the threshold, the corresponding data are determined as abnormal data:

(1) $$\mathrm{Anomaly}\;\mathrm{detection}=\left\{\begin{array}{c} \;\;\;\;\left|X_{t}-{\hat{X}}_{t}\right|>threshold,\;\;data\;is\;abnormal\\ \left|X_{t}-{\hat{X}}_{t}\right|\leq threshold,\;\;data\;is\;normal \end{array}\right.$$

where $X_{t}$ is input data and ${\hat{X}}_{t}$ is reconstruct input data.

### 3.2. LSTM-Autoencoder

**Figure 1:**
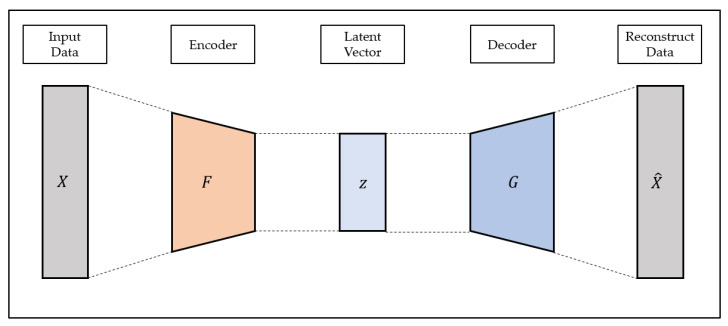
The structure of an autoencoder.

Autoencoder is a representative example of unsupervised learning that can efficiently learn the characteristics of input data. The Autoencoder consists of the encoder and decoder. The encoder generates latent variables using input data, and the decoder reconstructs the input data using latent vectors [56]. The structure of autoencoder is shown in Figure 1.

Since the autoencoder is a model that reconstructs the input data, the output of the autoencoder is referred to as the reconstructed data. The purpose of the autoencoder is to reconstruct input data and to prevent the input data from being output as is; various constraints such as the size limit of the latent vector are added to construct the model. The autoencoder model is trained in the direction of minimizing the reconstruction loss, which means the difference between the reconstruction data and the input data, as shown in Equation (2), and learns the most important characteristics from the input data to reconstruct the latent vector generated through the encoder with the decoder:

(2) $$L_{AE}=\frac{1}{n}\overset{n}{\underset{i}{\sum }}\left|X\left(i\right)-G\left(F\left(X\left(i\right)\right)\right)\right|$$

where $X\left(i\right)$ is input data, F is encoder operation of autoencoder and G is decoder operation of autoencoder.

Due to these characteristics, autoencoders are being used in various fields such as dimensionality reduction, feature extraction, and anomaly detection. However, in general, autoencoders are composed of multi-layered perceptron; thus, it is difficult to apply them to data with characteristics such as images and time series data.

**Figure 2:**
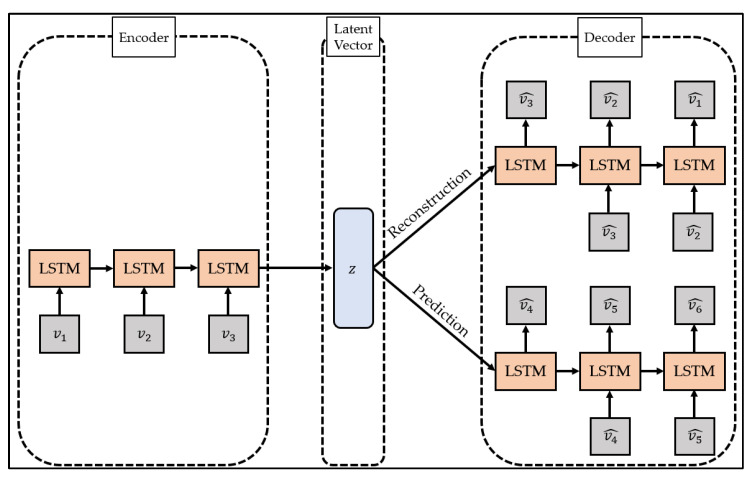
The structure of the LSTM-Autoencoder (composite model).

To overcome this limitation, studies are being conducted to change the models constituting the encoder and decoder of the autoencoder to suit the characteristics of the data. In particular, the LSTM-Autoencoder [51] proposed by Srivastava et al. configures the encoder and decoder of the autoencoder with LSTM, a type of recurrent neural network, and reconstructs the time series data by reflecting the characteristics of the time series data. The structure of LSTM-Autoencoder is shown in Figure 2.

The LSTM-Autoencoder consists of the encoder, reconstruction decoder, and prediction decoder. The encoder creates latent vectors by compressing time series data, the reconstruction decoder reconstructs them in reverse order, and the prediction decoder predicts future values from latent vectors. Unlike a general decoder, the reconstruction decoder reconstructs the input time series data in the reverse order. This is because low-range correlation is considered by reconstructing input time series data in reverse order, which makes optimization easier [51]. The LSTM-Autoencoder consists of one encoder and two decoders and has a structure different from that of general autoencoders. This is to overcome the limitations that may occur when the reconstruction decoder and the prediction decoder are trained [51]. If only the reconstruction decoder is trained, one limitation will be that overfitting may occur by preserving even trivial information from the time series data to generate a latent vector. In addition, when only the prediction decoder is trained, learning proceeds using only the latest information of the time series data, which may pose a problem since information from the past cannot be utilized. The LSTM-Autoencoder induces the model to store important information in the latent vector by simultaneously learning the reconstruction decoder and the prediction decoder and induces learning of information at all points in time series data.

### 3.3. Transfer Learning

**Figure 3:**
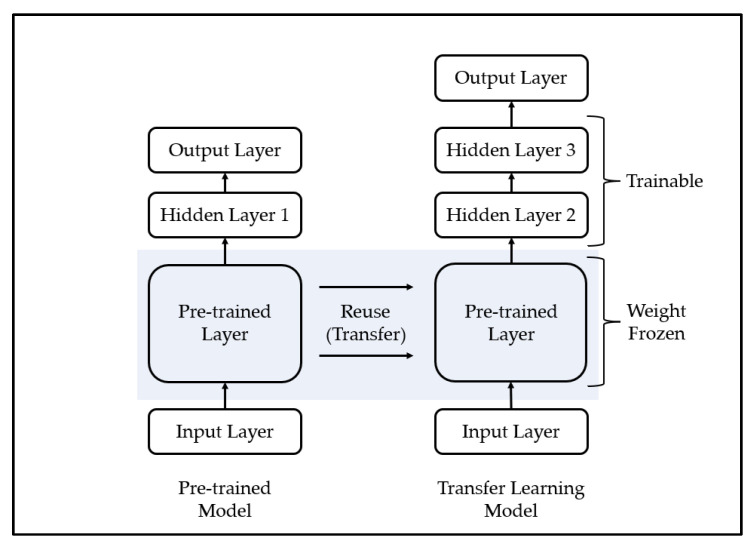
Example of Transfer Learning.

Transfer learning is a methodology applied to similar fields by using a model trained in a specific field and is mainly used for efficient training of deep learning models. A deep learning model is a methodology of predicting the future by modeling a pattern from training data, which is past data, and has been used in various fields with sufficient training data and generally demonstrated excellent performance [57]. However, one limitation of this methodology is that it yields poor performance when high-quality training data are not sufficiently collected, because it is difficult to model the complex pattern of the training data if high-quality training data are insufficient. To overcome these limitations, some recent studies have used transfer learning. The transfer learning methodology is shown in Figure 3.

The process of transfer learning is as follows. First, the deep learning model is trained using training data from other fields that show similar characteristics to a field in which it is difficult to collect training data. Second, it combines a part of the pre-trained model with a new deep learning model. In this case, since the high weight of the new deep learning model may disturb the weight of the pre-trained model, the weight of the pre-trained model is frozen. Third, learning is performed using a transfer learning model that consists of small-scale training data in areas where it is difficult to collect training data. Finally, if necessary, the weight freeze of the transfer learning model is released, and finetuning is performed using a small learning rate.

## 4. Materials and Methods

### 4.1. Dataset

**Figure 4:**
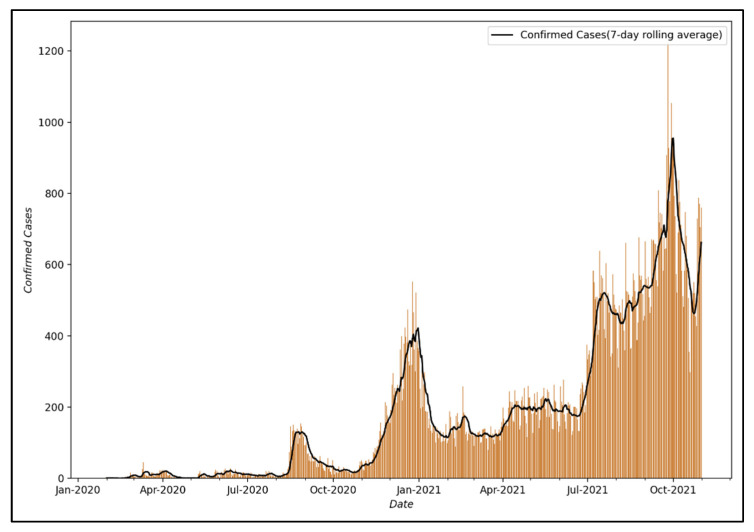
Number of confirmed COVID-19 cases in Seoul, South Korea (January 2020–October 2021).

This study used data on the daily number of confirmed COVID-19 cases by city and province provided by the Ministry of Health and Welfare of the Republic of Korea [58]. Among these data, Seoul was selected as the target city for analysis to perform modeling. It was judged that the data from Seoul were representative since Seoul is the capital of the South Korea and its commercial and residential districts are dense, with a large floating population vulnerable to infectious diseases, and the number of confirmed cases in Seoul has been increasing recently. Figure 4 shows the visualization of the number of confirmed COVID-19 cases from 24 January 2020, when the first confirmed case occurred in Seoul, to 31 October 2021.

Accordingly, the first analysis was conducted using a visualization of the number of confirmed COVID-19 cases from 24 January 2020, the date of the first confirmed case in Seoul, to 31 October 2021, and the following characteristics were identified. First, there is a pattern of a sharp drop in the number of confirmed COVID-19 cases on weekends or holidays. Regarding this pattern, it is judged that the number of confirmed cases is lower on weekends or holidays than on weekdays because the number of tests is reduced; thus, the pattern does not reflect the actual number of confirmed cases [59]. Second, it can be confirmed that despite the two surges in COVID-19 infections in 2020, the epidemic was successfully controlled. This is because South Korea implemented a social distancing policy, a type of NPI, to control the epidemic of COVID-19. As shown in Table 1, South Korea adopted social distancing measures as part of the effort to control the COVID-19 pandemic and ensured that the public health system does not collapse, and when the rapid surge in COVID-19 cases began, stronger social distancing measures such as “Social Distancing Level 2.5” were implemented.

In this study, the number of daily confirmed COVID-19 cases in Seoul was converted into a 7-day moving average based on the results of the primary analysis. This was performed to correct for the phenomenon that the number of confirmed cases becomes smaller due to a decrease in the number of testers on weekends or public holidays.

### 4.2. Method

In this study, anomaly detection and multi-step prediction were performed on the 7-day moving average of the number of confirmed COVID-19 cases in Seoul using the LSTM-Autoencoder model to study a decision support system for establishing and implementing PHSMs. The input of the LSTM-Autoencoder to be used in this study is the 7-day moving average of the number of confirmed COVID-19 cases for k days ($X_{t-k},\ldots ,X_{t-1}$), and the output is composed of the reconstruction decoder output and the prediction decoder output. In addition, based on these, when the effect of PHSMs varies, we propose a methodology that can efficiently learn it using transfer learning.

#### 4.2.1. Reconstruction Decoder

The reconstruction decoder generates output by reconstructing the 7-day moving average of the number of confirmed COVID-19 cases for k days in reverse order ($X_{t-1},\ldots ,X_{t-k}$). The encoder and reconstruction decoder of the LSTM-Autoencoder have similar structures with general autoencoders such as reconstructing input data, and these structures can be used in processes such as outlier detection.

As mentioned above, abnormal data in time series data refer to data that differ from past trends or patterns over time. The reconstruction decoder of the LSTM-Autoencoder proposed in this study is a model that trains using past trends or patterns by reflecting time-series characteristics and reconstructs input data. In this study, we intend to perform unsupervised anomaly detection on the COVID-19 epidemic at a specific time by utilizing the characteristics of the reconstruction decoder. When training using data on the COVID-19 epidemic at a specific time, the data on the effect of PHSMs implemented at that time are also included in the training. Performing anomaly detection using this reconstruction decoder can be seen as comparing the effect of PHSMs implemented at a specific time with the current effect of PHSMs. In this study, anomaly detection was used to compare and analyze the effects of PHSMs performed by period. In this case, the threshold, which is the criterion for determining abnormal data, was used as the maximum value of the loss function used in several previous studies:

(3) $$\mathrm{Anomaly}\;\mathrm{detection}=\left\{\begin{array}{c} \;\;\;\;\;\left|X_{t}-{\hat{X}}_{t}\right|>Loss_{Recon}^{max},\;\;\mathrm{date}\;\mathrm{is}\;\mathrm{abnormal}\\ \left|X_{t}-{\hat{X}}_{t}\right|\leq Loss_{Recon}^{max},\;\;\mathrm{date}\;\mathrm{is}\;\mathrm{normal} \end{array}\right.$$

where $X_{t}$ is input data and ${\hat{X}}_{t}$ is output of reconstruction decoder.

#### 4.2.2. Prediction Decoder

The prediction decoder predicts the 7-day moving average of the number of COVID-19-confirmed cases for the next k days ($X_{t},X_{t+1},\ldots ,X_{t+k-1}$) using a multi-step time series prediction strategy. There are various strategies for performing multi-step time series forecasting, but in general, the recursive multi-step forecast strategy and multiple output strategy are mainly used to perform multi-step time series forecasting.

The recursive multi-step forecast strategy is designed to perform multi-step time series forecasting by predicting the current step by receiving the step-forecast value from the previous k time as an input to the model after constructing a model that predicts one step and repeating it.

(4) $$\begin{array}{c} {\hat{X}}_{t}=model\left(X_{t-k},,\ldots ,X_{t-1}\right)\\ \vdots \\ {\hat{X}}_{t+k-1}=model\left({\hat{X}}_{t+k-2},{\hat{X}}_{t+k-3},\ldots ,X_{t-1}\right) \end{array}$$

Unlike the recursive multi-step forecast strategy that constructs a model that predicts one step, the multiple output strategy is a strategy that constructs a model that predicts multiples steps.

(5) $$\begin{array}{c} {\hat{X}}_{t},\ldots ,{\hat{X}}_{t+k-1}=model\left(X_{t-k},,\ldots ,X_{t-1}\right) \end{array}$$

Since the recursive multi-step forecast strategy constructs a model that predicts only one step, model training is performed smoothly. However, as in Equation (4), multi-step time series prediction operates recursively, so prediction errors accumulate, and the accuracy decreases as time series prediction is performed for a long period of time. On the other hand, the multiple output strategy performs stable multi-step time series prediction because the model predicts multiple steps at once without a recursive process. This study intends to perform stable multi-step time series prediction using a multiple output strategy.

#### 4.2.3. Transfer Learning

**Figure 5:**
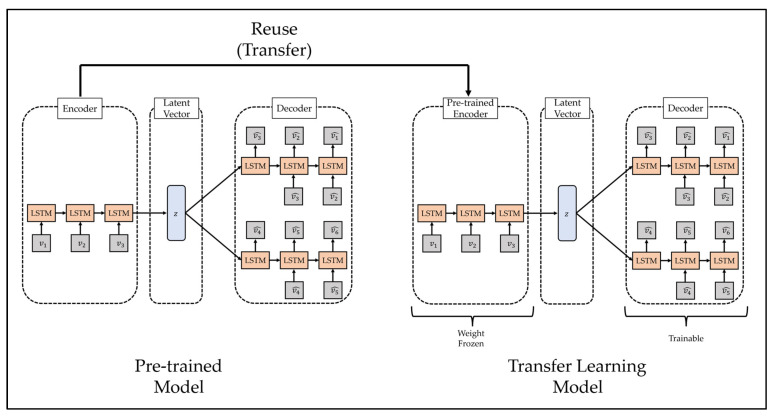
The transfer learning method applied in this study.

As discussed above, in this study, anomaly detection is performed using the reconstruction decoder of the LSTM-Autoencoder, and multi-step prediction is performed using the prediction decoder to study the decision support system for the establishment and implementation of PHSMs. However, the effectiveness of PHSMs can vary in response to internal or external factors. Furthermore, when the effect of the policy thus varies, the effectiveness of the proposed decision support system that learned the previous COVID-19 spread trend may be reduced depending on the policy direction of the country in which the variability occurs. To address this issue, we intend to improve the utility of the proposed decision support system by performing efficient training including the new epidemic trends by using the transfer learning methodology. The transfer learning methodology proposed in this study is shown in Figure 5.

The transfer learning process proposed in this study is as follows. First, the encoder part of the pre-trained LSTM-Autoencoder is combined with the reconstruction decoder and prediction decoder of the new LSTM-Autoencoder. Second, the transfer learning model is trained. Finally, if necessary, fine-tuning shall be performed. If transfer learning is thus used, training can be conducted more efficiently than training a new LSTM-Autoencoder, and its utility as a decision support system can be expected to increase.

#### 4.2.4. Summary of the Proposed Methodology

**Figure 6:**
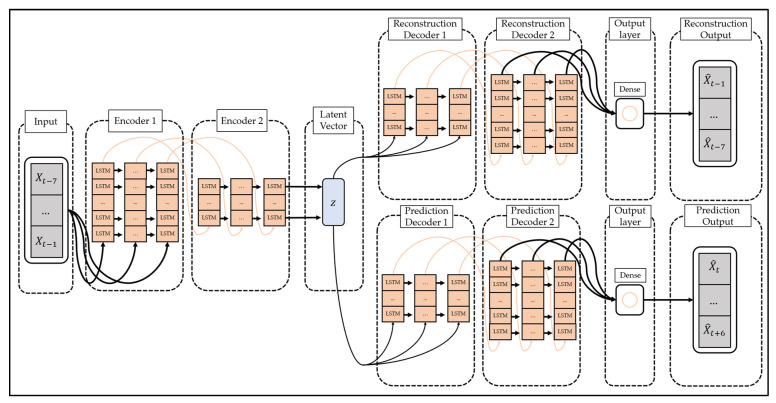
The structure of the LSTM-Autoencoder used in this study.

The LSTM-Autoencoder-based deep learning model proposed in this study receives the 7-day moving average value of the number of confirmed COVID-19 cases for 7 days as input and reconstructs it in reverse order, and then it predicts the number of confirmed COVID-19 cases for the upcoming 7 days. It is configured as shown in Figure 6 to predict the moving average value.

In addition, the LSTM-Autoencoder proposed in this study performed learning and evaluation of the basic model and the transfer learning model using data on the number of confirmed COVID-19 cases in a specific period to model the change in trend due to the effect of the policy. As mentioned above, the data on the number of confirmed COVID-19 cases in Seoul used in this study reflects the effects of PHSMs implemented in South Korea. A time-series deep learning model using these data also learns the effect of a policy implemented at a specific time during the learning process. Finally, we propose a basic model and transfer learning model that reflects the effects of PHSMs at a specific time by conducting the learning of the proposed LSTM-Autoencoder using the data on the number of confirmed COVID-19 cases in Seoul. The configuration of data used for training and testing by model is shown in Table 2.

The basic model was defined in this study as a model that trained the epidemic trend when only NPIs were used, among the various possible PHSMs. For this purpose, data from 24 January 2020 to 31 December 2020 were used as training data for the basic model, and from 1 January 2021 to 25 February 2021, they were used as test data for the basic model. In addition, the transfer learning model defined in this study is a model that trains the epidemic trend when the effect of PHSMs is varied due to various factors. For this purpose, data from 26 February 2021 to one month after the variation in the effect of PHSMs ($Abnormal\;Data_{t+1M}$) were used as training data. In addition, data from one month after the variation in the effect of PHSMs ($Abnormal\;Data_{t+1M}$) to two months after the variation in the effect of PHSMs ($Abnormal\;Data_{t+2M}$) were used as test data.

## 5. Results

### 5.1. Result of the Base Model

In this study, a decision support system for the establishment and implementation of LSTM-Autoencoder-based PHSMs was proposed. The experiment of the proposed decision support system was conducted in the Python 3.8 environment, and TensorFlow 2.3, a representative deep learning framework, was used to construct the deep learning model. LSTM-Autoencoder is a deep learning model and, in general, in order to ensure optimal performance of a deep learning model, a hyperparameter search must be performed and overfitting must be prevented. The hyperparameter search was performed to ensure the optimal performance of the LSTM-Autoencoder used in the proposed decision support system. In addition, to prevent overfitting, *L*1 and *L*2 weight regularizations and LSTM dropout [60] proposed by Gal et al., Layer normalization [61] proposed by Ba et al., and Early Stopping were applied. *L*1 and *L*2 weight regularization is a representative technique for prevent overfitting in machine learning. It prevents overfitting by adding a specific layer of weight regularization term to the loss function. The loss function to which the weight regularization term is added is shown in Equation (6):

(6) $$\begin{array}{c} Loss_{regularization}=Loss_{0}+\lambda_{L1}\left|W_{layer}\right|+\lambda_{L2}{\left(W_{layer}\right)}^{2} \end{array}$$

where $Loss_{0}$ is loss function, $W_{layer}$ is weight vector for a specific layer, $\lambda_{L1}$ is hyperparameter of *L*1 regularization and $\lambda_{L2}$ is hyperparameter of *L*2 regularization.

The deep learning model is trained in the direction of minimizing the loss function. In this process, it may have a large weight by sensitively responding to noise, which may cause overfitting. To solve this problem, weight regularization prevents overfitting by adding a weight regularization term using the concepts of *L*1 norm and *L*2 norm to the loss function. The results of model optimization are shown in Table 3.

**Figure 7:**
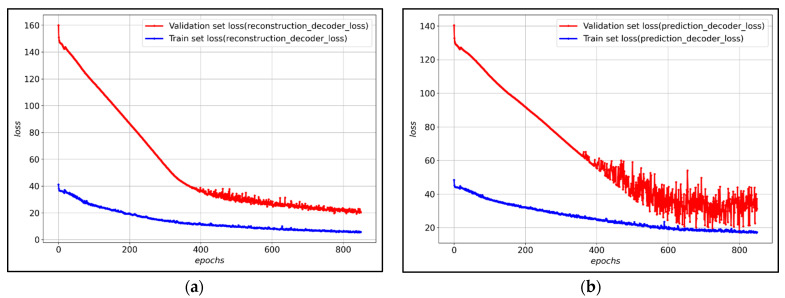
(**a**) Reconstruction decoder loss graph of the proposed LSTM-Autoencoder model; (**b**) Prediction decoder loss graph of the proposed LSTM-Autoencoder model.

Figure 7 shows the loss of training data and verification data for each decoder of the LSTM-Autoencoder to which the hyperparameter was applied. After about 800 epochs, validation loss converges, and it can be observed that both the reconstruction decoder and the prediction decoder trained stably.

Table 4 shows the detailed evaluation of the performance of the prediction decoder for predicting the 7 future days among the LSTM-Autoencoders proposed in this study. For the performance evaluation of the prediction decoder, Mean Absolute Error (MAE), Root Mean Squared Error (RMSE), and Mean Absolute Percentage Error (MAPE) evaluation indicators were used. In addition, in order to verify the superiority of the proposed prediction decoder compared to other models, statistical models ARIMA and ETS and deep learning models LSTM, DARNN [62] and TCN [63] were trained using the same data, and then the results were compared and verified. The results of the performance evaluation of the proposed LSTM-Autoencoder model confirmed that the prediction decoder of the LSTM-Autoencoder demonstrated superior performance compared to other models. This can be observed as the outcome of modeling long-term information as well by simultaneously learning the reconstruction decoder during the LSTM-Autoencoders prediction decoder learning process.

**Figure 8:**
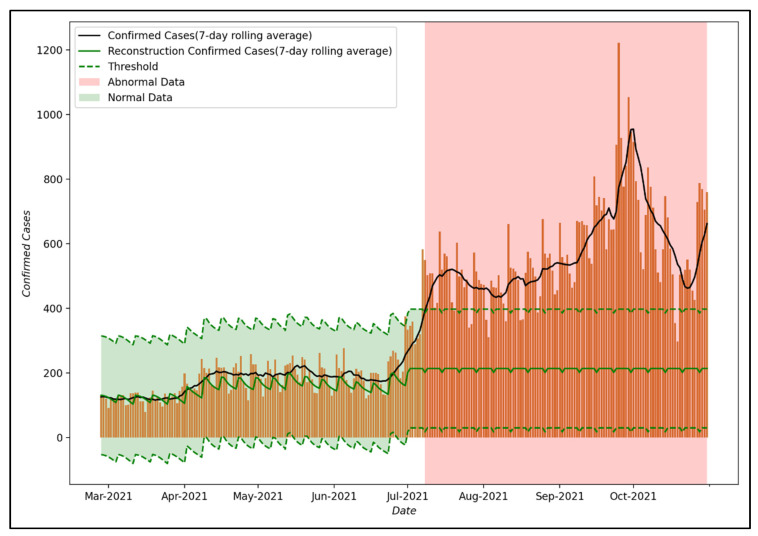
Result of anomaly detection using the base model.

Figure 8 shows the results of anomaly detection using the reconstruction decoder of the LSTM-Autoencoder model proposed in this study. As for the threshold, which is a measure to distinguish normal data from abnormal data, the maximum loss value of training data was used as in Equation (3). This can be interpreted as part of the effect of PHSMs, which the proposed LSTM-Autoencoder could not model when training the COVID-19 spread trend that reflected the effect of PHSMs. In this study, it is assumed that the effect of PHSMs varied when a larger error occurs compared to the threshold.

As the result of anomaly detection, up to July 2021, the reconstruction decoder was able to reconstruct the input data well, confirming that the number of confirmed COVID-19 cases in Seoul is within the threshold. However, after 8 July 2021, the reconstruction decoder did not reconstruct the input data well, such as convergence to a specific value, so it can be confirmed that the number of confirmed COVID-19 cases in Seoul is out of the threshold. This means that the trend in the COVID-19 epidemic after 8 July 2021 differs from that in 2020 and it suggests that the effect of PHSMs after 8 July 2021 differed compared to that of PHSMs in 2020 due to various factors.

### 5.2. Result of the Tranfer Learning Model

In Section 5.1 above, the results of anomaly detection of the base model verified that the effect of PHSMs changed after 8 July 2021. In this section, we tried to efficiently learn the effects of changed policies by using the data of the period determined by the base model as abnormal data as learning data and using transfer learning.

**Figure 9:**
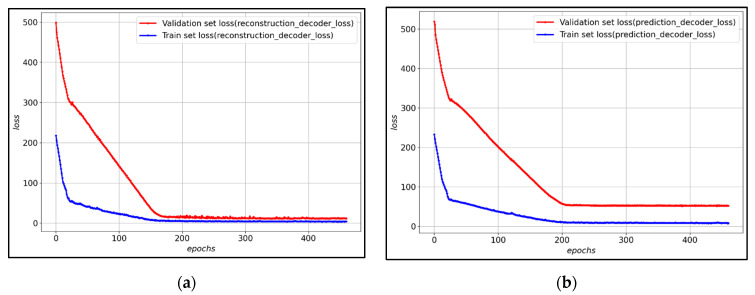
(**a**) Reconstruction decoder loss graph of the proposed transfer learning model; (**b**) Prediction decoder loss graph of the proposed transfer learning model.

The composition of the transfer learning model proposed in this study combines the encoder pre-trained in the base model and the new reconstruction decoder and prediction decoder. At this time, the weights of the pre-trained models were frozen because the high weights of the new reconstruction decoder and prediction decoder may disturb the weights of the pre-trained encoders. Figure 9 shows the loss of training data and verification data for each decoder of the transfer learning model. After about 200 epochs, the validation loss converges, and it is observed that both the reconstruction decoder and the prediction decoder are training stably.

Table 5 shows the detailed evaluation of the performance of the prediction decoder for predicting the future 7 days in the transfer learning model proposed in this study. For the performance evaluation of the prediction decoder, MAE, RMSE, and MAPE evaluation indicators were used. In addition, to verify the efficiency and strong performance of the proposed transfer learning model, it was compared and verified with the LSTM-Autoencoder model learned using the entire data set. This performance evaluation of the proposed transfer learning model confirmed that the prediction decoder of the transfer learning model performed better than the prediction decoder of the LSTM-Autoencoder trained using the entire data set. In addition, the loss converges in a relatively small number of epochs compared to the LSTM-Autoencoder of the proposed transfer learning model, and through this, the efficiency of the proposed model can be confirmed. However, we can identify that the MAE and RMSE values of the proposed transfer learning model are higher than that of the base model of Table 4. This is due to the fact that the scale of the epidemic during the period used for the learning of the transfer learning model is larger than the scale of the epidemic in the period used for the learning of the base model. This can be confirmed by comparing the MAPE values. The MAPE of the base model and the MAPE of the transfer learning model are similar, which indicates that the performance difference between the two models is not large.

## 6. Discussion

The COVID-19 pandemic is causing problems worldwide from a social, economic, and public health standpoint, and many countries have implemented PHSMs to overcome it. Since the establishment and implementation of these policies must be based on reasonable grounds, evidence-based studies applying a simulation approach are being conducted. However, most of these prior simulation-based studies had limitations in their utility as a decision support system for the establishment and implementation of PHSMs, such as a failure to reflect variations in policy effects and the short-term period of the simulations.

Therefore, this study proposed a decision support system based on LSTM-Autoencoder. The proposed decision support system attempted to overcome the limitations of models of the existing autoencoder by training the reconstruction decoder and prediction decoder at the same time. As a result of the experiment, it showed superior performance than the existing statistical models and deep learning models. It can also be interpreted as overcoming the limitations of the existing autoencoder as it shows superior performance relative to DARNN and TCN, which show high performance among time-series deep learning models.

The proposed decision support system predicted the moving average number of confirmed cases for the next 7 days when a policy was maintained. In addition, it was configured to provide information for policy re-establishment and implementation by comparing the effectiveness of the policies implemented during the two periods. And when the effect of PHSMs changed, transfer learning was performed to construct a model that can efficiently reflect the effect of the changed policy compared to the newly constructed simulation model. This is a factor that shows the superiority of the proposed decision support system compared to other simulation model-based decision support systems. Through this, we tried to increase the usability as a decision support system for policy establishment and implementation.

The decision support system proposed in this study has various advantages. First, the proposed decision support system is expected to be universally utilizable due to the characteristics of the data applied to training. The decision support system proposed in this study utilized the 7-day moving average number of COVID-19 cases in Seoul, South Korea, and these data are provided for public purposes in most countries. Therefore, it is expected that the proposed decision support system can be easily applied to other countries as well. In particular, it is judged that it can be easily applied to countries that have difficulties in establishing and implementing PHSMs because there is insufficient data to utilize the simulation approach. Therefore, the proposed decision support system is expected to have high usability.

Second, the proposed decision support system can provide a variety of information by quickly detecting changes in policy effects. As a result of anomaly detection of the decision support system proposed in this study, it was confirmed that the effect of PHSMs varied after July 8, 2021. During this period, the Delta variant was spreading in the Seoul metropolitan area, with the detection rate of the Delta variant significantly increasing from 4.5% to 12.7% compared to June [64]. In fact, the Delta variant (B.1.617.2) is known to have significantly higher infectivity and mortality compared to the existing COVID-19 [65]. The decision support system proposed in this study can be interpreted as quickly identifying the effect of PHSMs varies in response to the characteristics of the Delta variant. It is expected that the proposed decision support system will be able to provide a variety of information on variations in the effectiveness of policies quickly.

Finally, the proposed decision support system can be used flexibly according to policy directions. PHSMs can be strengthened or weakened depending on the policy direction of a given country. First, policy strengthening means restoring the effect of the varied policy by strengthening the policy when the effect of the PHSMs varies. In this case, it can be verified whether the effectiveness of the policy is restored through anomaly detection of the base model among the proposed decision support systems. On the other hand, weakening policies mean maintaining the effect of varied policies when the effect of PHSMs varies, which can be the course adopted when it is practically impossible to strengthen the policy due to social and economic factors. In this case, the effect of the varied policy can be efficiently trained by using the transfer learning model of the proposed decision support system. The decision support system proposed in this study can be used flexibly according to the policy direction and is expected to contribute to policy re-establishment and implementation.

## 7. Conclusions

Along with the rapid dissemination of COVID-19, the continuous emergence of major variants of the virus with high infectivity and lethality, such as the Alpha and Delta variants, and the consequent social and economic ripple effects are straining global resources. PHSMs are critically important for addressing such a severe situation, and research related to decision support systems to establish and implement PHSMs is also increasing. However, previous studies had limitations as a decision support system for policy establishment and implementation, due to their failure to reflect changes in the effectiveness of PHSMs and the short-term nature of their forecasts.

Therefore, this study proposed an enhanced decision support system for the establishment and implementation of PHSMs. The proposed decision support system focused on providing a reasonable basis for the establishment and implementation of PHSMs, such as promptly providing realistic information and information for maintaining policy continuity. Our experiment result confirmed that the loss of the LSTM-Autoencoder model proposed in this study was 1345.938% lower than that of the statistical model and 269.002% lower than that of the deep learning model. In particular, the 155.160% lower loss on average than DARNN and TCN, which show high performance among time series deep learning models, shows that the LSTM-Autoencoder proposed in this study trained the COVID-19 spread trend by reflecting the effect of PHSMs. In addition, the proposed transfer learning model has 240.828% lower loss with fewer epochs than the LSTM-Autoencoder trained over the entire period, confirming the efficiency and excellent performance of the proposed transfer learning model.

It is judged that the decision support system proposed in this study can be used for various purposes other than the reasonable establishment and implementation of PHSMs. First, the proposed decision support system is judged to be applicable to new infectious diseases. This applicability can be confirmed from the experimental results of the transfer learning method, which can efficiently learn the effect of a changed policy, as proposed in this study. Even after the current COVID-19 pandemic is over, a new epidemic of infectious disease may emerge, causing enormous social, economic, and public health damage as we have experienced during the COVID-19 pandemic. In the event of a new infectious disease epidemic, PHSMs will be used just as in the current COVID-19 pandemic, and if the decision support system proposed in this study is used, it is expected that reasonable establishment and implementation of PHSMs will be feasible at an early stage.

Second, it is judged that various policies other than PHSMs can be preemptively established by using the information generated from the proposed decision support system. Through the experimental results of the proposed decision support system, we confirmed that various information for decision making can be generated and changes in the effectiveness of PHSMs can be quickly identified. Such information can be used to preemptively establish various policies. As underscored above, the COVID-19 epidemic poses a significant threat to public health. The proposed decision support system is expected to be able to establish preemptive policies to maintain public health, such as the increase in beds for critically ill patients, by quickly detecting changes in the effects of PHSMs. Moreover, as mentioned above, the effectiveness of PHSMs may vary depending on the people’s compliance with policies. By disclosing the information generated by the proposed decision support system to the public, it will be possible to reduce the changes in the effectiveness of PHSMs by increasing the public’s policy compliance.

The decision support system proposed in this study offers various advantages, such as universal applicability, provision of rational and realistic information by quickly detecting changes in policy effects, and flexibility in the model for incorporating new policy directions. This confirms the superiority of the proposed decision support system. However, there are some limitations in relation to the analysis of changes in policy effects. First, it is possible to quickly identify variations in the effect of a policy, but there is a limit in identifying the cause of the change in the effect. Next, there is a limit in analyzing the degree to which the effect of a policy changes when such changes occur. These limitations are due to the fact that the LSTM-Autoencoder model used in this study is a deep learning-based model, and although these models show high prediction performance, they pose difficulties in analyzing a specific causal relationship. This shortcoming can be overcome by conducting additional research, such as research related to explainable artificial intelligence (XAI) and the design of quasi-experiments.

## Figures and Tables

**Table 1:** Changes in the status of social distancing levels by period in the South Korea.

Period	Levels of Social Distancing	Remarks
22 March 2020–5 May 2020	Enhancement Social Distancing	-
6 May 2020–15 August 2020	Distancing in Daily Life	
16 August 2020–29 August 2020	Social Distancing Level 2	Four levels of social distancing
30 August 2020–13 September 2020	Social Distancing Level 2.5	
14 September 2020–11 October 2020	Social Distancing Level 2	
12 October 2020–6 November 2020	Social Distancing Level 1	
7 November 2020–18 November 2020	Social Distancing Level 1	Five levels of social distancing
19 November 2020–23 November 2020	Social Distancing Level 1.5	
24 November 2020–7 December 2020	Social Distancing Level 2	
8 December 2020–23 December 2020	Social Distancing Level 2.5	
24 December 2020–3 January 2021	Social Distancing Level 2.5 ^1^	
3 January 2021–14 February 2021	Social Distancing Level 2.5	
15 February 2021–28 February 2021	Social Distancing Level 2	

^1^ Social distancing protocols are reinforced beyond Social Distancing Level 2.5, such as prohibiting private gatherings of 5 or more people.

**Table 2:** Configuration of data used for training and testing by model.

Model	Data	Period
Base model	Training data	24 January 2020–31 December 2020
	Test data	1 January 2021–25 February 2021
Transfer Learning model	Training data	$26\;\mathrm{February}\;2021–Abnormal\;Data_{t+1M}$
	Test data	$Abnormal\;Data_{t+1M}$ $–Abnormal\;Data_{t+2M}$

**Table 3:** Results of model optimization.

Hyperparameter	Layers	Value	Remark
LSTM Unit	Encoder 1	512	-
	Encoder 2	256	-
	Reconstruction Decoder 1	256	-
	Reconstruction Decoder 2	512	-
	Prediction Decoder 1	256	-
	Prediction Decoder 2	512	-
Weight regularization	Reconstruction Output layer	0.001	*L*1
		0.001	*L*2
	Prediction Output layer	0.05	*L*1
		0.005	*L*2
Dropout	Encoder 1 Encoder 2	0.2	-
	Reconstruction Decoder 1 Reconstruction Decoder 2	0.6	-
	Prediction Decoder 1 Prediction Decoder 2	0.4	-
Layer normalization	Encoder 1 Encoder 2 Reconstruction Decoder 1 Reconstruction Decoder 2 Prediction Decoder 1 Prediction Decoder 2	apply	-
Early Stopping		50	Patience
Learning rate	-	5 × 10^−5^	-
Batch size		8	-
Loss function	-	MAE (Mean Absolute Error)	-
Optimizer	-	Adam	-

**Table 4:** Evaluation of the proposed model.

Model		MAE	RMSE	MAPE
Proposed Model (LSTM-Autoencoder)	Prediction Decoder	17.172	25.368	10.960%
Statistical Models	ARIMA (Recursive Multi-step Forecast Strategy)	254.099	262.531	179.771%
	ETS (Multiple Output Strategy)	249.494	258.400	170.997%
Deep learning Models	LSTM (Multiple Output Strategy)	102.463	103.352	68.275%
	DARNN (Recursive Multi-step Forecast Strategy)	51.203	71.283	34.985%
	TCN (Multiple Output Strategy)	36.429	44.864	24.223%

**Table 5:** Evaluation of proposed model.

Model		Period Used for Training and Evaluations	MAE	RMSE	MAPE	Epoch
Proposed model (Transfer Learning Model)	Prediction Decoder	26 February 2021–9 September 2021	52.160	57.166	9.896%	416
LSTM-Autoencoder	Prediction Decoder	24 January 2020–9 September 2021	177.776	179.254	34.238%	1571

## Data Availability

The dataset used in this study is available at Corona 19 City/Province Status (https://www.data.go.kr/data/15043378/openapi.do (accessed on 31 December 2021)) of the Public Data portal.
